# An aggressive chondroblastoma of the knee treated with resection arthrodesis and limb lengthening using the Ilizarov technique

**DOI:** 10.1186/1749-799X-5-47

**Published:** 2010-07-28

**Authors:** Slavko Tomić, Aleksandar Lešić, Marko Bumbaširević, Jelena Sopta, Zoran Rakočević, Henry D Atkinson

**Affiliations:** 1 Special hospital for orthopaedic surgery "Banjica", Mihajla Avramovica 28, Belgrade 11000, Serbia; 2 Institute for Orthopaedic Surgery and Traumatology, Clinical Centre for Serbia, Višegradska 26, Belgrade 11000, Serbia; 3 Institute for Pathology, Belgrade School of Medicine, Belgrade 11000, Serbia; 4 Institute for Radiology, Clinical Centre for Serbia, Višegradska 26, Belgrade 11000, Serbia; 5 North London Sports Orthopaedics (NLSO), Department of Trauma and Orthopaedics, North Middlesex University Hospital, Sterling Way, London N18 1QX, UK

## Abstract

This case report describes the management of a 15 year old male with a biologically aggressive chondroblastoma of the knee. Following CT, bone scan, angiography and an open biopsy, the diagnosis was confirmed histologically and immunohistochemically. The patient underwent a 13 cm en-bloc excision of the knee, and knee arthrodesis with simultaneous bone transport using an Ilizarov ring fixator. Following 136 days of bone transport, the patient achieved radiological and clinical bony union after a total frame time of 372 days. He then commenced 50% partial weight-bear in a protective knee brace and gradually worked up to full weight-bearing by 4 months. The patient developed superficial pin tract infections around the k-wires on 2 occasions; these settled with a cephalosporin antibiotic spray and local dressings. At 13 years follow-up there are no signs of disease recurrence or failure at the fusion site. The patient is able to fully weight bear and stand independently on the operated leg. Knee arthrodesis with simultaneous limb-lengthening is an effective treatment modality following en-bloc resection of an aggressive chondroblastoma. The case is discussed with reference to the literature.

## Background

First described by Ewing in 1928, chondroblastomas were originally named "epiphyseal chondroblastomatous giant cell tumors of the proximal humerus" by Codman in 1931, and are often still termed Codman tumors [1-4]. They occur mostly in the second decade of life, and are more common in males [5-8]. Usually arising from the epiphyseal plate [9-11] and measuring between 1 and 7 cm [12], chondroblastomas are most frequently found in the proximal humerus, distal femur, proximal tibia, and the iliac bones [2-4,7,9,13-16]; they can also appear in the talus, ribs and digits [17-20].

Though normally benign, and accounting for 1-2% of all benign bone tumors [2-4], histologically aggressive forms of the disease can also occur [5,13,21,22], with associated high recurrence rates (5-38%) and occasional lung metastases [14,16,23].

We report the case of a biologically aggressive chondroblastoma of the knee treated with a 13 cm en-bloc excision, knee arthrodesis, and bone transport using an Ilizarov ring fixator.

## Case Presentation

**Figure 1:**
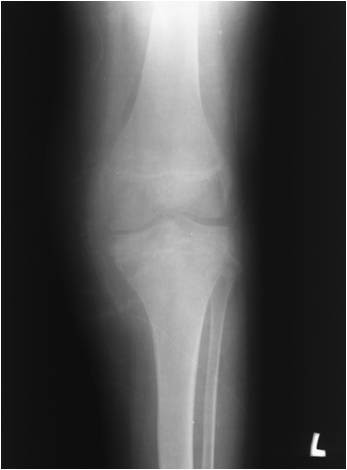
**Radiograph of the affected knee with an osteolytic lesion in the proximal tibia**.

**Figure 2:**
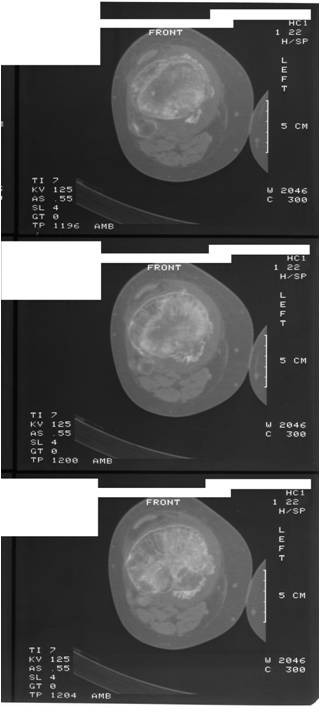
**CT scan demonstrating the lesion in the proximal tibia**.

In July 1995 a 15 year-old boy presented with a 6 month history of pain and swelling in the left knee. On examination he walked with an antalgic gait, there was a moderate left knee effusion, and the knee circumference was 5 cm greater than the right side. Range of movement was severely limited to 0 to 40 degrees of flexion. There was no local lymphadenopathy and he was constitutionally well. Plain radiographs (Figure 1) and CT scans (Figure 2) demonstrated an osteolytic process in the proximal left tibia, and a second lesion in the medial femoral condyle. Laboratory tests were within normal limits and a chest radiograph was normal. Angiography did not show any abnormal neovascularisation, and Technicum 99 bone scintigraphy showed a relative accumulation of radionucleotide in the proximal tibia.

**Figure 3:**
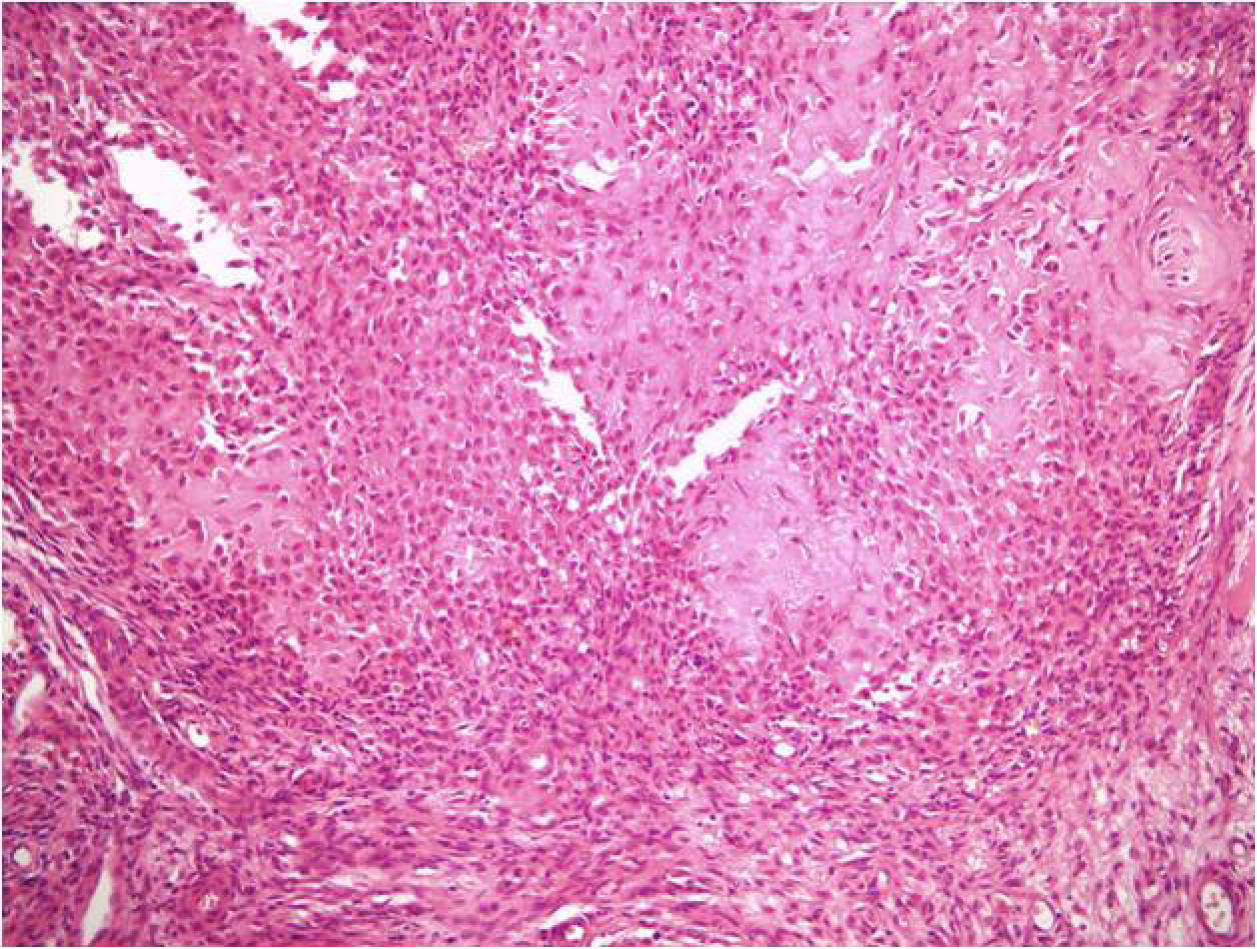
**Low power microscopy demonstrating a cellular lesion with chondroid stromal production and calcification assuming a fine linear pattern**. (HE stain, 40X)

**Figure 4:**
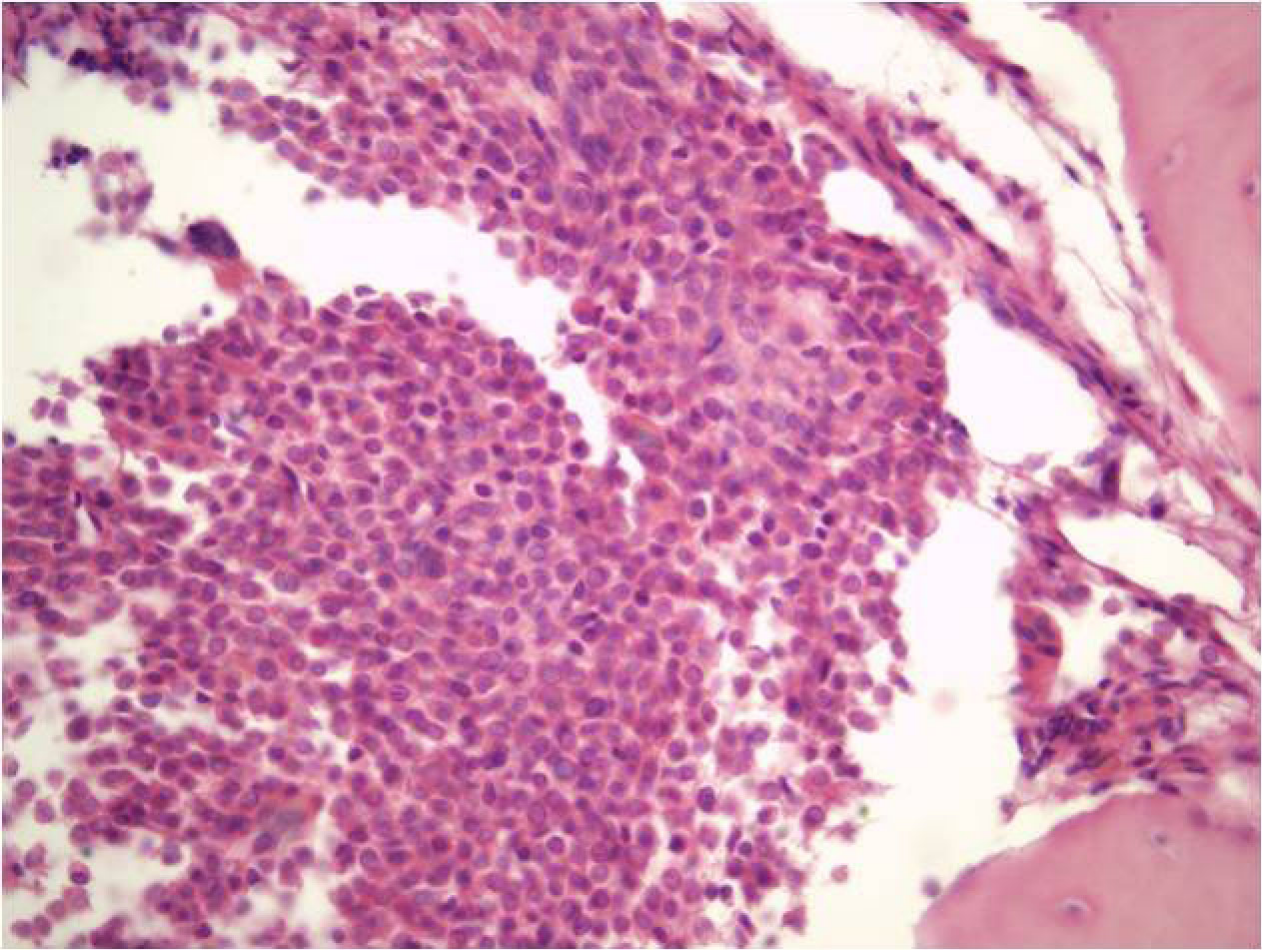
**High power microscopy showing polygonally-shaped mononuclear chondroblasts with an indistinct cytoplasm, and nuclei with central longitudinal grooves**. Multinucleated giant cells and mitoses are present. (HE stain, 200×)

**Figure 5:**
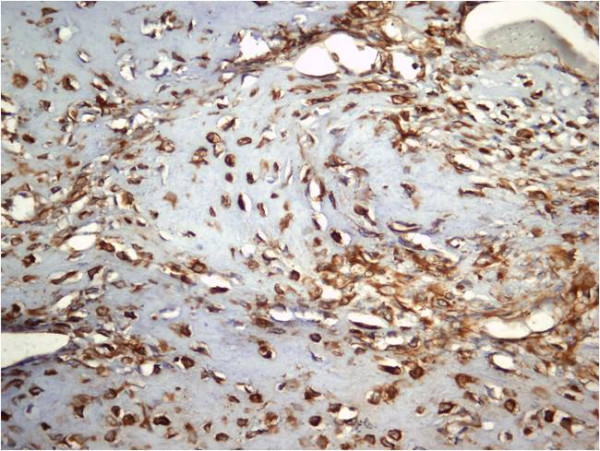
**Cells showing strong positivity for the S-100 protein**. (S-100 protein, 200×)

**Figure 6:**
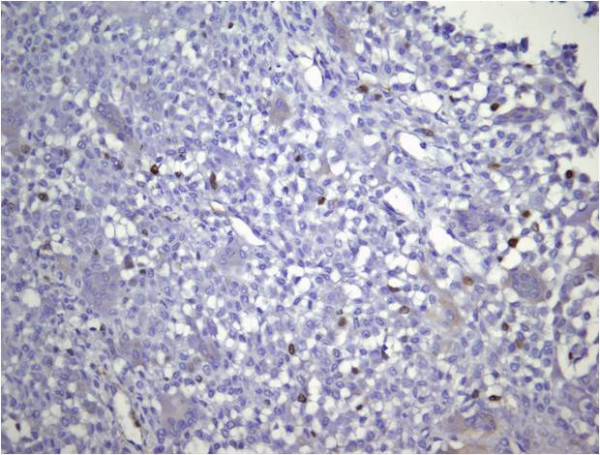
**Ki 67, proliferative factor is positive in 20% of cells**. (Ki 67, 100X)

The patient underwent open biopsy of the tibial lesion and microscopic histopathological analysis (HE stained and immunohistochemistry) confirmed an aggressive chondroblastoma (Figures 3, Figure 4, Figure 5, Figure 6). The tissue was composed of mononuclear polygon-shaped cells with a pink cytoplasm admixed with rare giant cells and chondroid stroma. The cells varied in both size and shape, with large nuclei, and were occasionally multinucleated. Up to 2 mitotic figures were present per high-power field. The cellular elements were separated by a scanty interstitial chondroid matrix with fine calcification arranged in a characteristic "chicken wire" pattern (Figures 3 and 4). The tumor cells showed a strong positivity for vimentin and S-100 protein. Proliferative factor Ki 67 was also positive in 20% of cells. (Figures 5 and 6).

**Figure 7:**
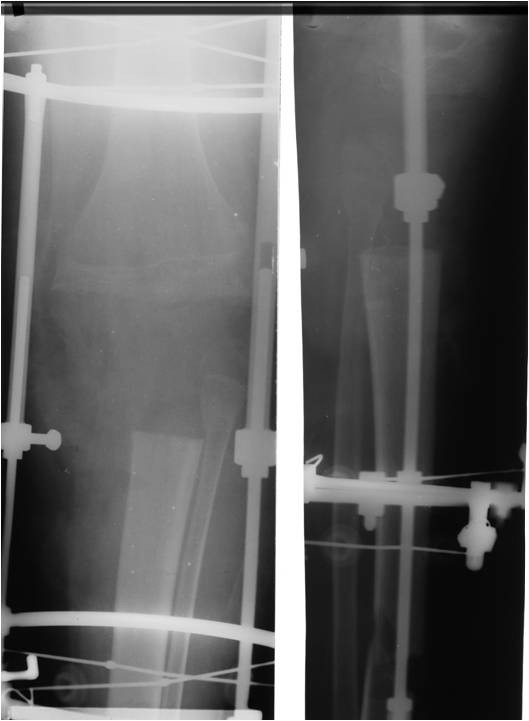
**Intraoperative radiograph following en-bloc bony resection**.

En-bloc resection of the knee was performed including the proximal 9 cm of tibia, tibial articular surface, the proximal fibula, the patella and quadriceps mechanism, the distal femoral articular surface and 3 cm of diseased femoral epiphysis. An Ilizarov frame was applied with one tibial fixation point 13 cm below the resection level and a second ring below the distal tibial metaphysis; the rings were each fixed with 3 Kirschner wires. The tibia was osteotomized between these 2 rings to allow for distractive proximal bone transport. A single ring was applied to the femur and was connected to the tibial rings using threaded rods (Figure 7). The soft tissues in the front of the knee were repaired in layers. The patient was allowed to weight-bear in his fixator.

**Figure 8:**
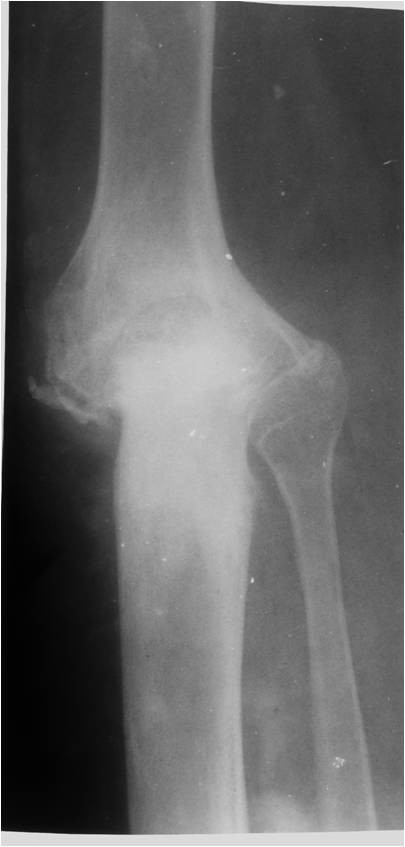
**AP radiograph showing bony union at the docking site**.

One week after surgery bone transport of the distal tibia was commenced at a rate of 1 mm per day. Distraction was continued for 136 days, with compression added every 10th day. There was only a single rest period of 10 days mid-transport due to peroneal nerve symptoms. These resolved without any long-term sequelae, and were thought to be due to the rate of distraction. Following docking of the proximal tibia with the distal femur, the patient had a 229-day consolidation period to allow for maturation of the regenerate bone (Figure 8); thus the Ilizarov frame was removed after a total of 372 days. The patient was then allowed to 50% partial weight-bear in a protective knee brace gradually working up to full weight-bearing at 4 months. Despite cleaning his pin-sites with soap and water every day the patient developed superficial pin tract infections around the k-wires on 2 occasions. These settled with a cephalosporin antibiotic spray and local dressings.

**Figure 9:**
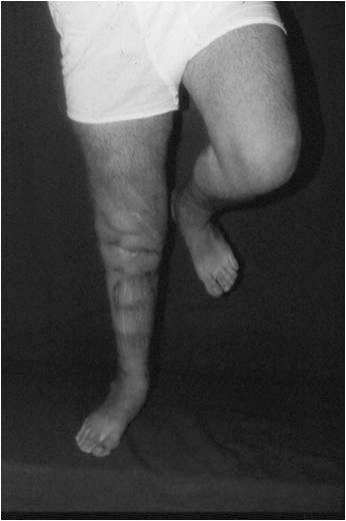
**Clinical photograph of the patient after 13 years**.

**Figure 10:**
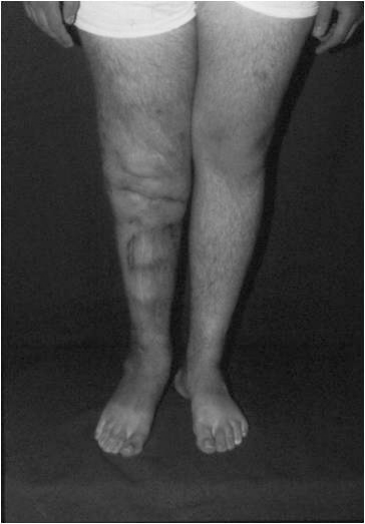
**Clinical photograph of the patient after 13 years**.

At 13 years follow-up there are no signs of disease recurrence or failure at the fusion site. The patient is able to fully weight bear and stand independently on the operated leg (Figures 9 and 10).

## Discussion

Benign chondroblastomas can often be treated with simple curettage with or without bone grafting [4,14], or with other adjuvant therapies including alcohol, cryotherapy and methylmethacrylate bone cementing [4,15], however these treatments are associated with recurrence rates of up to 30% [23], and are thus unacceptable in the more aggressive forms of this disease.

Aggressive disease requires an aggressive management strategy, and in cases involving the knee joint the treatment involves radical joint resection to prevent local recurrence and metastatic disease [5]. As with other tumors occurring around the knee, the residual defect can be managed with massive bony allograft or tumor prostheses following knee excision [13,24-26]. However these options are not always available due to financial constraints [27], and if the extensor mechanism has also been included in the resection then a mobile prosthesis is often not possible [28]. The bony defect can be alternatively managed through arthrodesis utilising a variety of internal fixation devices with bone graft/free fibula graft, or by external fixation in conjunction with bone transport in order to preserve limb length [15,21,29-33].

In a series of 8 patients undergoing resection arthrodesis for distal femoral giant cell tumors (GCT), successful union and good functional results were achieved in 7 patients for defects measuring 14-17 cm, using dual free fibular grafts and locked intramedullary nails, over a mean 14.5 months [33]. Another series achieved good functional outcomes and 100% union rates using dual fibular grafts alone following en bloc knee resection for 37 GCTs and 16 osteosarcomas, with defects ranging 9-24 cm [34]. A further report of 26 patients with primary bone tumors (including GCT, osteosarcoma and chondrosarcoma) underwent tumor resection and successful knee arthrodesis using autogenous bone graft [15].

Patients undergoing knee arthrodesis are often left with limb shortening particularly following large resections, and prior to skeletal maturity, and there are many advocates for performing simultaneous limb lengthening surgery [29,35-38]. The Ilizarov technique has been successfully utilised with bone transport in a series of 5 proximal tibial GCTs, with a mean defect of 5.7 cm [38], and in 7 distal femoral tumors with defects ranging from 8 to 20 cm [37]; others have also successfully used this technique in non-tumor cases, such as knee arthrodesis following infected total knee arthroplasty [39-42].

We favoured using the Ilizarov method with bone transport because of its versatility, its ability to provide excellent stability even with poor bone quality, the ability for our patient to fully weight-bear in his frame, and the predicted high rate of bony union [35,36,40-42]. In addition the technique creates "live" regenerate bone which we felt was preferable to "dead" allograft or non-vascularised fibular graft. However, aside from being technically challenging, this technique had the disadavantages of requiring a large proximal ring around the distal femur, which made walking awkward, and there was a prolonged fixation time of 372 days. Our patient also suffered pin tract infections on 2 occasions, which is common with all external fixation methods [37,40-44].

Though one might assume that a knee arthrodesis is an inferior treatment following knee joint excision, a comparison of patients undergoing knee arthrodesis, constrained total knee arthroplasty and below knee amputation, found that patients' function, walking velocity, efficiency and the rate of oxygen consumption were similar [21]. Arthrodesis patients had better limb stability and were able to perform more physically demanding activities, but had difficulty sitting. Arthroplasty patients had to be more sedentary due to weakness/instability, but were generally more positive [21]. Another study found that arthroplasty patients had better physical function scores, though arthrodesis patients had better mean pain scores and scored higher globally [45].

Our patient continues to do well 13 years following surgery, without any signs of disease recurrence or failure at the fusion site. He has no leg-length discrepancy, is able to fully weight-bear and stand independently on the operated leg, has no pain symptoms, and works full-time as a school teacher.

## Conclusion

In conclusion, knee arthrodesis with simultaneous limb-lengthening with an Ilizarov ring fixator is an effective treatment modality following en-bloc resection of an aggressive knee chondroblastoma. The technique is versatile, providing excellent stability, an ability to weight bear in the frame and has a predicatble high rate of bony union.

## Abbreviations

CT: Computed Tomography; HE: Hematoxylin and Eosin stain

## Competing interests

No competing interests or sources of funding declared

## Authors' contributions

ST, AL and MB managed and operated the patient. HDA, AL and MB wrote the manuscript. ST, JS and ZR assisted with the literature review and manuscript preparation. All authors have read and approved the final manuscript

## Consent

Written informed consent was obtained from the patient for publication of this case report and any accompanying images. A copy of the written consent is available for review by the Editor-in-Chief of this journal
